# ﻿Intersexuality in a natural population of the terrestrial isopod *Porcellioscaber*

**DOI:** 10.3897/zookeys.1101.77212

**Published:** 2022-05-18

**Authors:** Jens Zarka, Thomas Parmentier, Nicky Wybouw

**Affiliations:** 1 Terrestrial Ecology Unit (TEREC), Department of Biology, Ghent University, K.L. Ledeganckstraat 35, B-9000 Gent, Belgium Ghent University Gent Belgium; 2 Research Unit of Environmental and Evolutionary Biology, Namur Institute of Complex Systems, and Institute of Life, Earth, and the Environment, University of Namur, Rue de Bruxelles 61, 5000 Namur, Belgium University of Namur Namur Belgium

**Keywords:** Intersex phenotypes, Isopoda, sex-determination, *Wolbachia*

## Abstract

Intersex phenotypes are rarely observed in natural isopod populations and their expression is typically associated with infection of *Wolbachia*, a reproductive parasite that manipulates arthropod reproduction. During an intensive sampling effort of a natural population of the isopod *Porcellioscaber*, an adult individual was isolated that expressed both male and female traits. The intersex individual exhibited clearly developed external male genitalia and carried multiple eggs in its brood pouch. No *Wolbachia* infection could be identified in this individual, a result that needs to be approached with caution due to suboptimal DNA preservation for diagnostic PCR assays. *Wolbachia* were, however, detected in two adult females of the same population, and appear closely related to isolates that infect other terrestrial isopod species. This is the first demonstration that intersex phenotypes can arise under natural conditions in *P.scaber*.

## ﻿Introduction

Sex-determination mechanisms regulate the sexual differentiation of organisms and are highly diverse across the animal kingdom. Sex-specific differentiation can rely on external environmental cues but can also be solely regulated by the segregation of genetic factors (Bull 1985; Zarkower 2001). In a wide range of arthropod species, sex-determination is controlled by multiple genetic factors (Cordaux et al. 2011; Moore and Roberts 2013). For instance, populations of the house fly *Muscadomestica* carry several male-determining chromosomes and an additional female sex-determination locus (Dübendorfer et al. 2002; Hediger et al. 2010). Intersex individuals that express both male and female traits spontaneously arise at low frequencies in natural populations of certain arthropod species (Narita et al. 2010). In isopods, the appearance of intersex individuals is often linked to infection of *Wolbachia*, probably one of the most widespread invertebrate-associated bacteria (Bouchon et al. 1998, 2009; Engelstädter and Hurst 2009; Cordaux et al. 2011, 2012). *Wolbachia* are maternally transmitted endosymbiotic bacteria that infect the reproductive tissues of arthropods and nematodes (Werren 1997). *Wolbachia* spread within host populations by manipulating host reproduction in multiple ways (Engelstädter and Hurst 2009). In isopods, *Wolbachia* can feminize genetic males into functional phenotypic females (Rigaud et al. 1991; Bouchon et al. 1998; Cordaux et al. 2004). However, under certain conditions, *Wolbachia*-mediated feminization can be incomplete and intersex individuals can arise (O’Neill et al. 1997; Herran et al. 2020).

To date, *Wolbachia*-infected individuals have been described in at least 39 isopod species, with all *Wolbachia* isolates belonging to the *Wolbachia* B- or F-supergroup (Bouchon et al. 1998; Cordaux et al. 2001; Zimmermann et al. 2021). *Wolbachia*-mediated feminization of the common pill woodlouse *Armadillidiumvulgare* is well understood and has become a model system to study the evolution of sex-determination pathways (Rigaud et al. 1997; Herran et al. 2020). Here, the androgenic hormone synthesized by the androgenic gland is responsible for the formation of male gonads. It has been experimentally shown that the implantation of an androgenic gland into females can induce functional sex reversal depending on the timing of implantation (Suzuki and Yamasaki 1997). These findings indicate that all *A.vulgare* individuals possess the necessary genetic basis that is required for male and female sexual differentiation (Rigaud et al. 1997).

In genetic males, *Wolbachia* likely inhibit the development of the androgenic gland by either targeting the androgenic hormone promotor or the androgenic hormone receptor hereby feminizing the individual (Rigaud et al. 1997). Incomplete feminization has been associated with low densities of *Wolbachia* during embryonal development (O’Neill et al. 1997; Bouchon et al. 2009; Cordaux et al. 2011; Herran et al. 2020). Low *Wolbachia* densities can be the result of increased temperature levels as has been shown for *A.vulgare* (Herran et al. 2020). Despite the widespread occurrence of *Wolbachia* in natural isopod populations (Bouchon et al. 1998, 2009), intersex individuals have been observed in only a limited number of isopod species, including *A.vulgare*, *Armadillidiumalbum*, *Porcelliolaevis*, *Sphaeromarugicauda* and *Ligiaoceanica* (Juchault et al. 1991; Bouchon et al. 1998; Ben Nasr et al. 2010).

Here, we present the first record of an intersex *Porcellioscaber* collected from a natural population in Snellegem (Belgium) in August 2020. The individual carried a large number of eggs in its brood pouch, and can thus be considered as a functional female. However, the individual also possessed clearly developed external male genitalia.

## ﻿Materials and methods

### ﻿Isopod collection

We used cuboid pitfalls (25 cm × 7.5 cm × 8 cm) containing an approximately 1 cm bottom layer of plaster to collect isopods in Brugge, Snellegem, and Vleteren (Parmentier et al. 2021). The plaster was moisturized to prevent the desiccation of all trapped isopods, so that we could collect the isopods alive; all isopods were collected one week after installation of the pitfalls and preserved on 70% ethanol. Individuals were preserved on 70% ethanol for three months before phenotyping.

### ﻿Diagnostic PCR detection of *Wolbachia* infection

In addition to the intersex individual, we also isolated four adult females that exhibited normal sexual differentiation from the Snellegem population. Sterility was maintained by working in a biological safety cabinet. After washing the specimens twice in sterile water for 1 min, DNA was extracted from whole bodies using the Quick-DNA Universal kit (BaseClear, the Netherlands). DNA integrity was tested by amplifying a fragment of cytochrome c oxidase subunit I (*COI*) using the LCO1490 and HC02198 primers (Folmer et al. 1994). A standardized PCR approach was performed to test *Wolbachia* infection in the five *P.scaber* samples (one intersex individual and four normal females) using DreamTaq DNA Polymerase (Life Technologies Europe BV). The standard primers of the multilocus sequence typing system for *Wolbachia* were used to amplify fragments of the *wsp*, *ftsZ*, *hcpA*, *coxA*, and *gatB* genes, standard molecular markers to detect *Wolbachia* infection (Baldo et al. 2006). *Wolbachia*-infected *Myrmicascabrinodis* workers were used as positive controls. The *hcpA* gene fragment was Sanger sequenced for one of the *Wolbachia*-infected *P.scaber* females that exhibited normal sexual differentiation (MACROGEN Europe B.V.).

## ﻿Results

**Figure 1.**
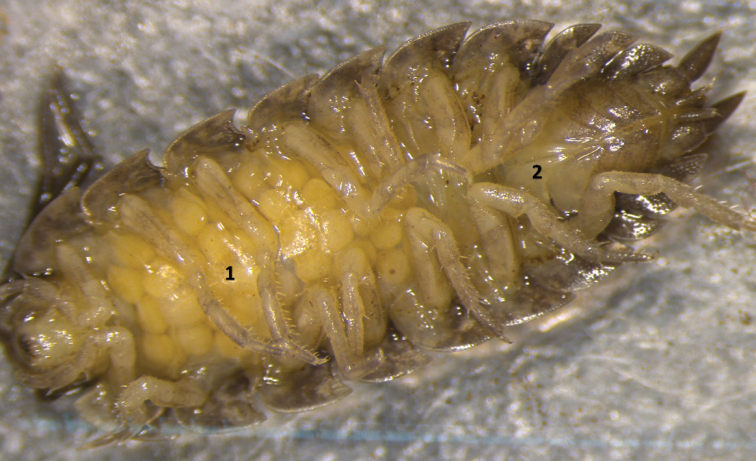
Ventral view of the intersex specimen of *P.scaber* with multiple eggs in the marsupium (**1**) and a male pleon (**2**).

**Figure 2.**
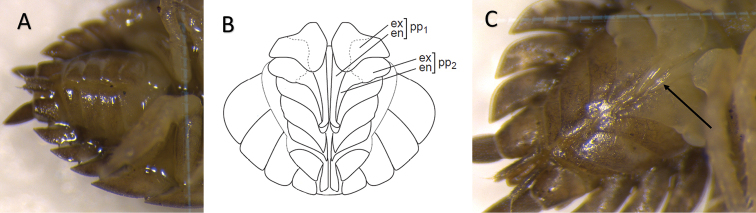
**A** ventral view of the pleon of a normal female *P.scaber***B** drawing of the ventral view of the male pleon of *A.vulgare*, similar in morphology to *P.scaber***C** ventral view of the pleon of intersex *P.scaber* with the endopodite of the first pleopod marked with an arrow. Abbreviations: **pp1** first pleopod; **pp2** second pleopod; **ex** exopodite; **en** endopodite (**B** drawn from Shultz 2018).

From a collection of 7,814 individuals, we found a *P.scaber* individual in the Snellegem population that carried eggs and, although egg viability was not ascertained, could be considered as a functional female (Fig. 1). However, in contrast to female isopods (Fig. 2A), this individual possessed an elongated and well-developed endopodite of the first pleopod, a canonical male sexual characteristic in isopods (Fig. 2C). In addition, the exopodites and endopodites of the first and second pleopods were also shaped differently than females, forming a male pleon (Fig. 2B, C). In the marsupium of the individual multiple eggs were found, comparable in number to normal females. We did not manage to amplify a fragment of the *COI* gene for the intersex individual, while PCR amplification was successful for four normal Snellegem females that were collected at the same time. This apparent discrepancy in DNA integrity might be due to a longer exposure to air for the intersex individual during photography. Using diagnostic PCR assays, we were unable to identify a *Wolbachia* infection of the intersex individual. *Wolbachia* infection was confirmed in two of the four normal females. Sanger sequencing of the *hcpA* gene fragment revealed a 100% identity to a *Wolbachia* isolate that was previously retrieved from the isopod *Helleriabrevicornis* (Sicard et al. 2014). The partial *hcpA* gene sequence was deposited in the GenBank database under the accession number OM459769.

## ﻿Discussion

Intersex individuals are rarely observed in natural populations of arthropods (Narita et al. 2010). The intersex phenotype of the current study appears to be present at an extremely low frequency in natural populations of *P.scaber*. However, additional intersex individuals were possibly overlooked, because they were not carrying visible eggs at the time of collection. One study previously described a similar, but not identical, intersex phenotype in *P.scaber* where three specimens displayed greatly reduced male genitalia and were expected to be functional females according to the authors (Sassaman and Garthwaite 1984). In contrast to our individual, these individuals came from laboratory stocks and not from a wild natural population. In addition, our individual did not show reduced genitalia.

It is tempting to speculate that incomplete *Wolbachia*-mediated feminization caused the intersex phenotype in this individual. *Wolbachia* are widespread in *P.scaber*, infecting populations across Europe (Bouchon et al. 1998). We uncovered that the natural Snellegem population was also infected. Previous work has shown that both males and females of *P.scaber* carry *Wolbachia* (Bouchon et al. 1998). Moreover, interspecific transfer of feminizing *Wolbachia* into *P.scaber* revealed that its sex-determination mechanisms can be manipulated by the reproductive parasite and can result in intersex individuals under controlled laboratory conditions (Bouchon et al. 1998). However, it remains uncertain whether the *Wolbachia* variants that naturally infect *P.scaber* are able to feminize genetic males. Unfortunately, we could not bring more clarity to this outstanding question due to our inability to confirm *Wolbachia* infection in our intersex individual. Due to the suboptimal preservation of the individual (Marquina et al. 2021), DNA degradation was likely too severe and interfered with our diagnostic PCR assays, a hypothesis that is supported by our inability to amplify a *COI* gene fragment. Currently, we cannot exclude that other mechanisms, such as pollution- or virus-induced developmental abnormalities, caused the formation of a functional female *P.scaber* with male genitalia (Juchault et al. 1991; Ford 2012).
